# Inflamed Eyelids

**Published:** 1891-10

**Authors:** 


### INFLAMED EYELIDS.

This complaint is known by the red appearance of the free margins of the eyelids. It is in reality an inflammation of the hair tubes of the lids. It is a disease more of childhood than of adult life, but it may affect persons of all ages. It is seen more in delicate and scrofulous children than in robust ones, and is often a sign of a weakly constitution. It is sometimes left by measles or other fever of childhood. But the most common cause of it is impure air, which irritates the lids and so starts it off. Where dust, acid vapors or smoke mingles with the air of the room, there we expect to find most cases. It is a common disease among the lower classes in Ireland and Scotland, where the smoke from peat fires mixes with the air in the cabin or hut before escaping from the open door or by a hole in the roof. There is a slight redness of the free margins of the lids, a little matter is poured out and glues the hairs together, at night the hairs of the upper and lower lids unite and glue up the lids, the presence of the little crusts of matter may also cause irritation. If the complaint becomes chronic from want of proper treatment then the hairs may fall from the lids and cause baldness of the lids. This complaint is not a dangerous one, but if well marked it makes the sufferer look unsightly. The treatment for this complaint must be chiefly constitutional. Have only three meals a day ; do not eat flesh meat more than once a day, and not always that. Avoid all rich, fatty, greasy and sweet foods and drinks. Take two or three hours' exercise every day in the open air ; children cannot be out too much. Be sure to have the purest air possible in all your rooms ; if the chimney smokes have it made right. Keep the windows open according to the season, but never less than two inches night and day in all weathers. Avoid all close and unventilated places, and keep the blinds up at night. Have a weekly warm bath or wash all over. The lids themselves may be smeared with a little unsalted lard at night-time to prevent them from sticking together; and the eyes may be bathed with milk and warm water twice a day. If these directions are carried out, the complaint will gradually disappear without having to use golden or other ointment usually prescribed by drug doctors. The great secret is to be sure to get the purest air possible both night and day.

